# Effect of Misalignment between Successive Corneal Videokeratography Maps on the Repeatability of Topography Data

**DOI:** 10.1371/journal.pone.0139541

**Published:** 2015-11-23

**Authors:** FangJun Bao, JunJie Wang, JinHai Huang, Ye Yu, ManLi Deng, LinNa Li, AYong Yu, QinMei Wang, Pinakin Gunvant Davey, Ahmed Elsheikh

**Affiliations:** 1 The Affiliated Eye Hospital of WenZhou Medical University, Wenzhou, 325027, China; 2 The institution of ocular biomechanics, Wenzhou Medical University, Wenzhou, Zhejiang Province 325027, China; 3 School of Engineering, University of Liverpool, Liverpool L69 3GH, United Kingdom; 4 College of Optometry, Western University of Health Sciences, Pomona, CA 91766, United States of America; 5 NIHR Biomedical Research Centre for Ophthalmology, Moorfields Eye Hospital NHS Foundation Trust and UCL Institute of Ophthalmology, United Kingdom; Bascom Palmer Eye Institute, University of Miami School of Medicine, UNITED STATES

## Abstract

**Purpose:**

To improve the reliability of corneal topographic data through the development of a method to estimate the magnitude of misalignment between successive corneal videokeratography (VK) maps and eliminate the effect of misalignment on the repeatability of topography data.

**Methods:**

Anterior and posterior topography maps were recorded twice for 124 healthy eyes of 124 participants using a Pentacam, and the repeatability of measurements was assessed by calculating the differences in elevation between each two sets of data. The repeatability of measurements was re-assessed following the determination of the magnitude of misalignment components (translational displacements: x_0_, y_0_ and z_0_, and rotational displacements: α, β and γ) between each two data sets and using them to modify the second data set within each pair based on an Iterative Closest Point (ICP) algorithm. The method simultaneously considered the anterior and posterior maps taken for the same eye since they were assumed to have the same set of misalignment components. A new parameter, named Combined Misalignment parameter (CM), has been developed to combine the effect of all six misalignment components on topography data and so enable study of the association between misalignment and the data repeatability test results.

**Results:**

The repeatability tests resulted in average root mean square (RMS) differences in elevation data of 8.46±2.75 μm before ICP map matching when simultaneously considering anterior and posterior surfaces. With map matching and misalignment correction, the differences decreased to 7.28±2.58 μm (P = 0.00). When applied to only the anterior maps, misalignment correction led to a more pronounced reduction in elevation data differences from 4.58±1.84 μm to 2.97±1.29 μm (P = 0.00). CM was found to be associated with the repeatability error (P = 0.00), with posterior maps being responsible for most of the error due to their relatively lower accuracy compared to anterior maps.

**Conclusions:**

The ICP algorithm can be used to estimate, and effectively correct for, the potential misalignment between successive corneal videokeratography maps.

## Introduction

The transparent cornea is a most important optical component of the outer ocular tunic, contributing about 70% of the total refractive power of the eye [1]. The reliable characterisation of corneal shape is critical for the assessment of vision quality and has become increasingly necessary for several applications, particularly in planning refractive surgery procedures [2],[3], diagnosis and management of corneal disorders [4],[5] and fitting of contact lenses [6],[7] and especially for orthokeratology corrections. In these applications and in order to monitor the corneal condition longitudinally, it is essential that topography mapping is accurate and repeatable.

Repeated measurements, either within the same setting or over time, are unlikely to be taken from precisely the same angle and position and the potential misalignment between readings will likely have an effect in inflating the differences between topography measurements and the errors in repeatability tests [8]. In addressing this point, the current study employs an Iterative Closest Point (ICP) algorithm (a dominant method for registration of 3D free-form surfaces [9],[10]) to quantify and correct for misalignment between successive maps. The ICP algorithm can accurately align views of an object (collected from different viewpoints and therefore located in separate coordinate systems) to obtain a larger map of the object’s surface. Applying this technique to successive corneal maps enables estimation of relative misalignment and the application of rigid-body transformation to eliminate the effect of this misalignment.

Commonly, repeatability of videokeratography (VK) maps is tested at *single points* using measures such as the within-subject standard deviation (Sw), within-subject coefficient of variation (CV), coefficient of repeatability (CoR), intraclass correlation coefficients (ICCs) and Bland Altman Plots [11–17]. To widen the effectiveness of these measures, they can be applied repeatedly at points across the part of the corneal surface covered by VK maps. While repeating this exercise enables a more comprehensive evaluation of topographic data repeatability, it makes the analysis more expensive and time consuming, and still not truly representative of the whole map area. In this study, repeatability is assessed through direct comparisons between the elevation data obtained across the whole VK map area, allowing a more comprehensive representation of data repeatability and assessment of the distribution of data mismatch between successive maps.

The determination of misalignment components (translational displacements: x_0_, y_0_, z_0_, and rotational displacements: α, β, γ) using ICP algorithm allows relative, rigid-body transformation of maps to eliminate misalignment and hence enable testing data repeatability before and after the transformation. However, while this exercise can provide qualitative assessment of the effect of misalignment on data repeatability, the fact that misalignment is determined in the form of six independent components makes it challenging to have a quantitative correlation between misalignment and data repeatability. This desired outcome is thought to be quite important clinically as it could provide a direct relation between the misalignment between maps (which can be quantified using ICP) and the reliability of topographic data used to guide clinical decisions. For this reason, this study attempted to create a parameter that describes the overall effect of misalignment and is therefore dependent on the six misalignment components and their individual effects on data repeatability.

The current study concentrated on using the Pentacam topographer (Oculus Optikgerate GmbH, Wetzlar, Germany), which offered all data needed for analysis, namely both anterior and posterior surface maps, elevation data in the form of x, y, z coordinates and reliable, repeatable measurements of biometric parameters such as the anterior chamber depth (ACD), central corneal thickness (CCT), corneal curvature (CC) and best-fit sphere (BFS) [11–13, 18]. However, the study findings are independent of the imaging method used by the Pentacam, and therefore should be applicable to topography maps produced by other VK techniques.

## Material and Methods

### Study participants

124 healthy subjects (78 male and 46 female) aged between 18 and 40 years (mean 23.2±4.3 years) were recruited from corneal refractive surgery patients and medical trainees of the Eye Hospital of Wenzhou Medical University, China. The study followed the tenets of the Declaration of Helsinki and was approved by the Scientific Committee of the Eye Hospital. Signed informed consent was obtained from the subjects prior to conducting the procedures. The inclusion criteria were ocular astigmatism less than 2.00D, intraocular pressure less than 21 mmHg, no ocular disease, no history of trauma, no previous ocular surgery and no contact lens wear for at least two and four weeks before topography measurement for soft contact lens and gas permeable contact lens (RGP) wearers, respectively. Eyes that did not meet these criteria were excluded.

### Data Acquisition

The study parameters included refractive error (RE), corneal curvature in horizontal (Kh) and vertical directions (Kv), central corneal thickness (CCT), and corneal elevation data of anterior and posterior surfaces. RE was measured with a phoroptor (RT-2100, Nidek Inc, Gamagori, Japan) and converted to spherical equivalent, SE. Kh, Kv, CCT and corneal elevation were provided by a Pentacam. Room lights were switched off during data acquisition. Each subject was asked to blink just before each measurement and fixate on a light inside the machine. After each acquisition, the device was moved back and realigned for the next scan. The measurements continued until two scans with an instrument-generated quality factor of at least 95% and 90% were obtained for the anterior and posterior surfaces, respectively. Only data from the right eyes were collected and used in analysis. All measurements were taken by the same trained examiner (JC). The elevation maps of each anterior and posterior surface were exported in the form of a matrix with 141×141 grid.

### Repeatability analysis

The root mean square (RMS) of the difference in elevation data between two successive topography measurements, and within their area of overlap, was used as a measure of the repeatability of topography data. In this study, the RMS of elevation data difference was calculated for each pair of measurement both before and after eliminating the effect of misalignment components estimated by the ICP algorithm. In addition to providing a single measure of data match, the results of the elevation comparisons enabled assessment of the distribution of error across the topography measurement area.

The area of overlap between the two measurements after data transformation was also calculated as an additional measure of repeatability, although in this case it was much less effective since the area of overlap did not undergo significant reduction even with the largest misalignments observed in the study.

### Topography analysis using ICP

As a feature-based registration and surface matching technique, ICP is directly applicable to the featureful 3D shape of the corneal anterior and posterior surfaces. The algorithm checks the similarities between the overlapping maps to determine the rigid-body transformations needed for the best possible match. Since its introduction by Yang and Paul [9, 10], the ICP algorithm has become the dominant method for image registration, and subsequent studies have improved the original algorithm and the stability of its analysis procedure [19]. The variant of ICP algorithm used in this paper is presented in this section.

**Fig 1:**
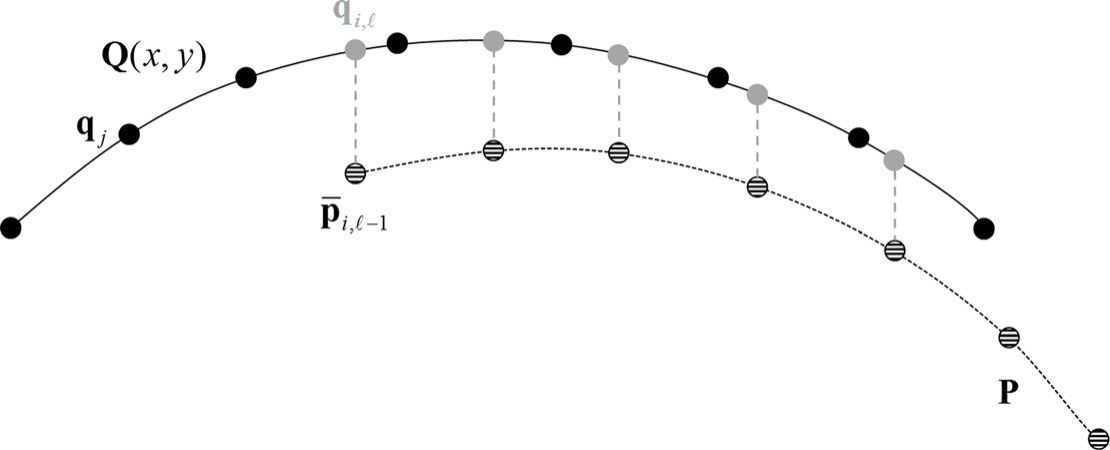
Method adopted to locate points $q_{i,l}$ on surface Q that correspond to points ${\bar{p}}_{i,l-1}$ on surface P in the lth iteration. Hashed points ${\bar{p}}_{i,l-1}$ (although moving through iterations) and black points **q**_*j*_ are original discrete points of the measurements of surface P and surface Q. Surface Q has been fitted to set of Zernike polynomials and expressed by Q(*x*,*y*) and thus represented in figure as a solid curve. Point $q_{i,l}$ in grey corresponds to, and has the same x and y coordinates as, ${\bar{p}}_{i,l-1}$ and hence is not necessarily one of the original discrete points **q**_*j*_.

In the ICP algorithm, a spatial transformation of a surface **P** (second topography map) is conducted to maximize its match with another surface **Q** (first topography map), with both surfaces expressed as sets of discrete data points (**p**_**i**_ ∈ **P;*i*** = 1,2,**…,m**) and (**q**_**j**_ ∈ **Q;*j*** = 1,2,**…,n**), and *m* and *n* being the numbers of points on the two surfaces, respectively. The spatial transformation takes the form of a rigid-body transformation **T** = (**R|t**), where **R** = **R**_**z**_ **R**_**y**_ **R**_**x**_ is the spatial extrinsic rotation around the origin of a fixed coordinate system, consisting of three sequential rotations R_x,_ R_y,_and R_z,_ about *x*, *y* and *z* axes by rotational components α, β and γ:

$$R_{x}=\left(\begin{array}{ccc} 1 & 0 & 0\\ 0 & \cos\alpha & -\sin\alpha \\ 0 & \sin\alpha & \cos\alpha \end{array}\right),R_{y}=\left(\begin{array}{ccc} \cos\beta & 0 & \sin\beta \\ 0 & 1 & 0\\ -\sin\beta & 0 & \cos\beta \end{array}\right),R_{z}=\left(\begin{array}{ccc} \cos\gamma & -\sin\gamma & 0\\ \sin\gamma & \cos\gamma & 0\\ 0 & 0 & 1 \end{array}\right)$$ (1)

and **t** = (**x**_0_ **y**_0_ **z**_0_)^**T**^ is the translation vector including translational components along *x*, *y* and *z* axes. The transformation is applied to points **p**_**i**_ in the form: $\bar{p_{i}}=Rp_{i}+t$, and the registration problem then focuses on finding the transformation (**R**, **t**) that provides the best possible alignment of surface **P** with surface **Q**. This is done by iteratively updating the position of surface **P** such that in the lth iteration

$$f(R_{l},t_{l})=\sum_{i=1}^{m_{l}}{\left\|n_{i,l}^{T}({\bar{p}}_{i,l}-q_{i,l})\right\|}^{2}$$ (2)

is minimised, resulting in ${\bar{p}}_{i,l}={\hat{R}}_{l}{\bar{p}}_{i,l-1}+{\hat{t}}_{l}$ with ${\hat{R}}_{l}$ and ${\hat{t}}_{l}$ being the optimal solution for the minimisation of Eq (2). In this calculation, $q_{i,l}$, now also subscripted by ***i*** rather than ***j***, is the corresponding point or correspondence of ${\bar{p}}_{i,l-1}$ (i.e. the point on surface **P** that have been updated by the last iteration), determined before the minimisation of Eq (2) by intersecting surface **Q** with a vertical line that originates from ${\bar{p}}_{i,l-1}$ (Fig 1). Further, $n_{i,l}$ is the normal to surface **Q** at point $q_{i,l}$ and therefore $n_{i,l}^{T}({\bar{p}}_{i,l}-q_{i,l})$ is the point-to-plane distance, which is the perpendicular distance from ${\bar{p}}_{i,l}$ to the tangential plane to surface **Q** at point $q_{i,l}$. Instead of Euclidean distance between ${\bar{p}}_{i,l}$ and $q_{i,l}$, the ICP algorithm minimises the point-to-plane distance, which allows surface **P** to slide over surface **Q** more efficiently and leads to better convergence [19].

A consequence of this technique is that the correspondence $q_{i,l}$ is not necessarily one of the original discrete points **q**_***j***_ representing surface **Q** and also the number of points included in Eq (2) may be less than the number of points in the original measurements surface **P** and surface **Q** (e.g. $m_{l}\leq m$) because some of the points may have moved outside the region of surface **Q** (e.g. Fig 1). This makes it necessary to fit points **q**_***j***_ to orthogonal Zernike polynomials $\left(Q\left(x,y\right)=\sum_{k=1}^{\infty }a_{k}z_{k}(x,y)\right)$ and use the polynomials and the coordinates x, y of point ${\bar{p}}_{i,l-1}$ to locate point $q_{i,l}$ and compute $n_{i,l}$ on surface **Q**. Zernike polynomials are widely used in ocular applications including wave-front representation and topography fitting [20], and in practice a finite set of Zernike polynomials is sufficient to reconstruct **Q**(*x*,*y*) such that

$$Q(x,y)=\sum_{k=1}^{M}a_{k}Z_{k}(x,y)+\varepsilon$$ (3)

where *M* is the number of Zernike polynomials determined by the polynomials’ order *N* such that *M* = (*N*+1)(N+2)/2 and *ε* represents the truncation error. The polynomial coefficients ***a***_***k***_ may be obtained by linear regression onto the measured discrete opography data [21]. The order of Zernike polynomials in this paper has been chosen as 10 (i.e. *N* = 10), for which the reconstruction error, i.e. the root mean square (RMS) error between points on the original map and the reconstructed map, has consistently been less than 1 μm.

The matching process starts with a reasonable estimate **T**_0_ = (**R**_0_ | **t**_0_) of the position of surface **P**, which in this study was zero values for all six misalignment components. This estimate brings surface **P** to its initial position ${\bar{p}}_{i,0}=R_{0}p_{i}+t_{0}$ and the iterative process given earlier is implemented until the left hand term in Eq (2) reaches a stable value.

The outcome of this process is an estimate of the misalignment components between surface **P** and surface **Q** in the form of rigid-body rotations, **R**, and displacements, **t**. In addition, the process results in a shifted surface $\bar{P}$ with points ${\bar{p}}_{i}=Rp_{i}+t$ which have been corrected for misalignment with surface **Q**. This overall transformation **T** = (**R | t**) is computed after the ICP process has reached the stable solution (e.g. after *L* iterations) by a recursive relation expressed as

$$R={\hat{R}}_{L}\cdots {\hat{R}}_{1}R_{0}\;\mathrm{and}\;t={\hat{R}}_{L}\cdots {\hat{R}}_{1}t_{0}+{\hat{R}}_{L}\cdots {\hat{R}}_{2}{\hat{t}}_{1}+\cdots +{\hat{R}}_{L}{\hat{t}}_{L-1}+{\hat{t}}_{L}$$ (4)

In this study, it is expected that misalignment between topography measurements would affect anterior and posterior maps equally. Therefore, the ICP process adopted combined both maps in the objective function in the form:

$$f(R_{l},t_{l})=\sum_{i_{a}=1}^{m_{a,l}}{\left\|n_{i_{a},l}^{T}({\bar{p}}_{i_{a},l}-q_{i_{a},l})\right\|}^{2}+\sum_{i_{p}=1}^{m_{p,l}}{\left\|n_{i_{p},l}^{T}({\bar{p}}_{i_{p},l}-q_{i_{p},l})\right\|}^{2}$$ (5)

where the points on anterior and posterior maps are indexed by *i*_*a*_ and *i*_*p*_ respectively and the numbers of both maps are $m_{a,l}$ and $m_{p,l}$ respectively. This process ensured that only one set of misalignment components was obtained for each eye.

Repeatability of the topography data was calculated both before and after the ICP process as the RMS elevation differences between maps P and Q for each participant, i.e. $RMS_{1}=\sqrt{\frac{\sum_{i=1}^{m_{1}}{\left(p_{z,i}-q_{z,i}\right)}^{2}}{m_{1}}}$ and $RMS_{2}=\sqrt{\frac{\sum_{i=1}^{m_{2}}{\left({\bar{p}}_{z,i}-{\bar{q}}_{z,i}\right)}^{2}}{m_{2}}}$ where **p**_*z*,*i*_ and **q**_*z*,*i*_ are z coordinates of points **p**_*i*_ and **q**_*i*_ before ICP process, ${\bar{p}}_{z,i}$ and ${\bar{q}}_{z,i}$ are z coordinates of ${\bar{p}}_{i}$ and ${\bar{q}}_{i}$ after ICP process, respectively. ${\bar{q}}_{i}$ is the corresponding point of ${\bar{p}}_{i}$ located after the original points **p**_*i*_ have been corrected for misalignment with surface **Q** (Fig 1). This calculation considers only data points in the overlapping region of the two topography maps, and the numbers of points in the two maps within the overlapping region are *m*_1_ and *m*_2_, respectively.

### ICP validation

In order to assess the validity of the ICP outcome, the study started with an exercise in which the ability of the algorithm to predict pre-set misalignments between two identical corneal surfaces is tested. The exercise relied on surface **Q**; an 8mm diameter topography map of a randomly selected participant in the form of a 141×141 rectangular grid with x, y, z coordinates at each point. Surface **P** was then generated using the same VK data, but after introducing pre-determined translational and rotational transformations. Surface **P** did not share the same x, y coordinates with surface **Q** as a result of the transformations introduced, but retained the noise that typically existed in the measurements. Eleven cases with the misalignment components depicted in Table 1 were included in the study. The cases referred to the averages and standard deviations of misalignment components determined for the clinical dataset of the study; x_0-average_ = 0.76±49.14 μm, y_0-average_ = 1.05±53.92 μm, z_0-average_ = -0.45±0.93 μm, α_average_ = -0.01±0.41 degrees, β_average_ = 0.01±0.37 degrees and γ_average_ = 0.04±2.35 degrees. The 11 cases considered a change in an individual misalignment component or simultaneous changes in 2, 4 or all 6 components. The ability of the ICP algorithm to estimate the known misalignments and to transform surface **P** to fit surface **Q** was used to validate the algorithm before using it with the clinical data of this study.

**Table 1:** Pre-set misalignment components used in 11 cases considered in the validation study.

	x_0_	y_0_	z_0_	α	β	γ
Case 1	A	A	A	A	A	A
Case 2	A+1.96×SD	A	A	A	A	A
Case 3	A	A+1.96×SD	A	A	A	A
Case 4	A	A	A+1.96×SD	A	A	A
Case 5	A	A	A	A+1.96×SD	A	A
Case 6	A	A	A	A	A+1.96×SD	A
Case 7	A	A	A	A	A	A+1.96×SD
Case 8	A+1.96×SD	A+1.96×SD	A	A	A	A
Case 9	A	A	A	A+1.96×SD	A+1.96×SD	A
Case 10	A+1.96×SD	A	A+1.96×SD	A+1.96×SD	A	A+1.96×SD
Case 11	A+1.96×SD	A+1.96×SD	A+1.96×SD	A+1.96×SD	A+1.96×SD	A+1.96×SD

* A = mean value, SD = standard deviation

### Combined misalignment parameter

The fact that misalignment is expressed in 6 independent components makes it difficult to directly assess the relative importance of one misalignment component over another or the combined effect of all components on the repeatability of topographic data. In order to address this point, a new parameter named the combined misalignment parameter (CM) has been developed to combine the effect of the 6 misalignment components on map matching and potentially present a clear measure of data repeatability. The derivation of a value for the new misalignment parameter in a particular case involved the application of the 6 misalignment components obtained using the ICP algorithm on an ellipsoidal topography map $\frac{x^{2}}{a^{2}}+\frac{y^{2}}{b^{2}}+\frac{{\left(z-d\right)}^{2}}{c^{2}}=1$ with average dimensions; a = 8.65, b = 8.54, c = 9.54, d = -9.54 and radius 9mm (obtained from best fit ellipsoids with study topography maps). As described above, the application of the 6 misalignment components resulted in a rigid-body transformation of the map: ${\bar{s}}_{i}=Rs_{i}+t$, where **s**_*i*_ were sample points on the ellipsoidal map, ***i*** = 1,2,……***m*** and *m* was number of points. A set of new points ${\bar{\bar{s}}}_{i}$ on the original ellipsoidal topography map were then computed using the same x and y coordinates as for ${\bar{s}}_{i}$ to enable the computation of the combined misalignment parameter as: $CM=\sqrt{\frac{\sum_{i=1}^{m}{\left({\bar{\bar{z}}}_{i}-{\bar{z}}_{i}\right)}^{2}}{m}}$ where ${\bar{\bar{z}}}_{i}$ and ${\bar{z}}_{i}$ are the z coordinates of ${\bar{\bar{s}}}_{i}$ and ${\bar{s}}_{i}$, respectively. CM, which combined the effect of the six misalignment components, was used in the study to enable direct assessment of the association between the magnitude of misalignment and the result of the repeatability tests.

### Statistical analysis

The fit with normal distribution was tested through a single sample K-S check test. Column diagram plots were drawn to show the distribution of the misalignment components. The comparison of misalignment components with zero was tested in the One-Sample T Test. Commercial software SPSS 20.0 (Chicago, USA) was utilized for all analyses and a two-tailed probability of P < 0.05 was considered statistically significant.

## Results

### Validation study

The success of the ICP algorithm in estimating the pre-set misalignment components of the 11 cases described in Table 1 is illustrated in the results presented Table 2. The max error was -4.35% of the pre-set values and all the ratios between the errors and the corresponding pre-set values were on average equal to 0.43%, with a standard deviation ratio of 1.05%.

**Table 2:** Matching results of the cases considered in the validation study.

		x_0_, μm	y_0_, μm	z_0_, μm	α, degree	β, degree	γ, degree
Case 1	Pre-set	0.759	1.048	-0.445	-0.013	0.005	0.042
	Computed	0.758	1.045	-0.445	-0.013	0.005	0.041
	Diff Ratio	-0.17%	-0.25%	-0.02%	-0.11%	-0.30%	-1.02%
Case 2	Pre-set	97.078	1.048	-0.445	-0.013	0.005	0.042
	Computed	97.074	1.020	-0.445	-0.012	0.005	0.040
	Diff Ratio	-0.01%	-2.67%	-0.05%	-1.57%	-0.70%	-3.37%
Case 3	Pre-set	0.759	106.723	-0.445	-0.013	0.005	0.042
	Computed	0.726	106.710	-0.445	-0.013	0.005	0.043
	Diff Ratio	-4.35%	-0.01%	-0.07%	-0.76%	-4.34%	2.44%
Case 4	Pre-set	0.759	1.048	1.383	-0.013	0.005	0.042
	Computed	0.758	1.045	1.383	-0.013	0.005	0.041
	Diff Ratio	-0.17%	-0.25%	0.01%	-0.11%	-0.30%	-1.02%
Case 5	Pre-set	0.759	1.048	-0.445	0.781	0.005	0.042
	Computed	0.755	1.042	-0.446	0.781	0.005	0.041
	Diff Ratio	-0.62%	-0.55%	0.02%	0.01%	-0.76%	-0.75%
Case 6	Pre-set	0.759	1.048	-0.445	-0.013	0.731	0.042
	Computed	0.751	1.046	-0.445	-0.013	0.731	0.041
	Diff Ratio	-1.05%	-0.21%	-0.04%	-0.13%	-0.01%	-0.50%
Case 7	Pre-set	0.759	1.048	-0.445	-0.013	0.005	4.655
	Computed	0.764	1.045	-0.445	-0.013	0.005	4.655
	Diff Ratio	0.66%	-0.24%	-0.04%	-0.09%	0.53%	-0.01%
Case 8	Pre-set	97.078	106.723	-0.445	-0.013	0.005	0.042
	Computed	97.075	106.703	-0.445	-0.013	0.005	0.041
	Diff Ratio	-0.003%	-0.02%	-0.04%	-1.13%	-0.88%	-2.51%
Case 9	Pre-set	0.759	1.048	-0.445	0.781	0.731	0.042
	Computed	0.750	1.056	-0.445	0.781	0.731	0.042
	Diff Ratio	-1.28%	0.79%	0.003%	-0.01%	-0.01%	0.15%
Case 10	Pre-set	97.078	1.048	1.383	0.781	0.005	4.655
	Computed	97.079	1.038	1.387	0.781	0.005	4.654
	Diff Ratio	0.002%	-0.98%	0.29%	0.0%	-0.10%	-0.01%
Case 11	Pre-set	97.078	106.723	1.383	0.781	0.731	4.655
	Computed	97.086	106.699	1.383	0.781	0.731	4.655
	Diff Ratio	0.01%	-0.02%	0.002%	0.02%	0.01%	-0.002%

x_0_, y_0_, z_0_ represent the translational displacements of corneal surface; α, β, γ represent the angular rotations about the three main axes (x, y and z); *Pre-set* means the artificial misalignments added to the corneal elevation data pre topography matching; *Computed* means the misalignments calculated using the ICP algorithm; *Diff Ratio* in percentages means the ratio of the difference compared with Pre-set misalignments

The ICP success was highest with z_0_ misalignments, where the average error ratio was 0.006±0.097%. The error ratio became larger, yet still negligible, with x_0_, y_0_, α, β and γ misalignments with similar error ratios of -0.63±1.35%, -0.40±0.86%, -0.35±0.55%, -0.62±1.30%, and -0.60±1.50% respectively.

### Study of clinical data

**Fig 2:**
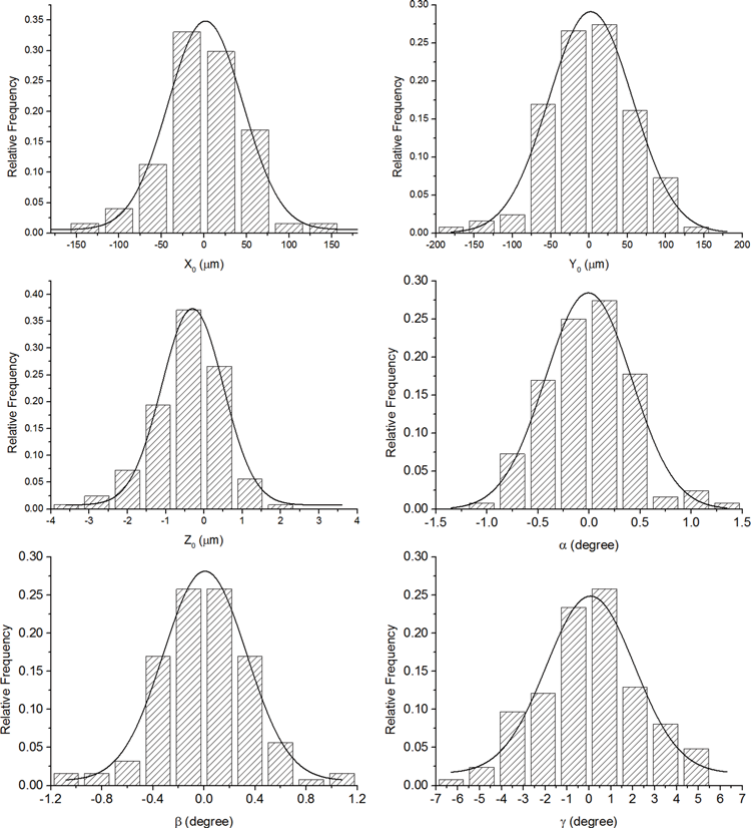
Column diagrams of the distribution of the six misalignment components (x_0_, y_0_, z_0_, α, β, γ) as calculated using the ICP algorithm for all clinical data.

There was a wide range of RE (0.13 to -11.13D), K_h_ (39.52 to 46.71 D), Kv (40.18 to 48.04 D) and CCT (463 to 607 μm). Averages, standard deviations and ranges of misalignment components x_0_, y_0_, z_0_, α, β, γ were 0.76±49.14 μm (-151.82–156.58), 1.05±53.92 μm (-181.02–125.36), -0.45±0.93 μm (-3.99–1.86), -0.01±0.41 degrees (-0.99–1.29), 0.01±0.37 degrees (-1.1–1.15), 0.04±2.35 degrees (-6.2–5.49), respectively. All misalignment components between successive measurements were normally distributed as demonstrated in Fig 2, and most (x_0_, y_0_, α, β, γ) were not significant from zero except for z_0_ (P = 0.000). Before topography matching, the repeatability test was conducted on successive maps and the RMS of fit errors was 8.46±2.75 μm (3.91–19.89 μm) for both anterior and posterior surfaces, considered simultaneously. The RMS of fit errors then decreased to 7.28±2.58 μm (2.52–17.19 μm) (reduction of 13.95%) following the ICP correction of misalignment. The correction of misalignment was associated with a negligible change in the area of overlap within each pair of topography maps; from 98.17±0.22% to 98.00±0.20% of total area. Due to the expected lower accuracy of posterior maps compared to anterior maps, the map matching exercise was repeated while considering only anterior maps then only posterior maps. With anterior maps, the RMS of fit errors reduced significantly from 4.58±1.84 μm (1.79–10.90 μm) to 2.97±1.29 μm (1.08–7.63 μm) (reduction of 35.13%) following correction for misalignment, while the change was from 11.40±4.05 μm (4.95–28.83 μm) to 10.25±3.89 μm (3.11–25.05 μm) (reduction of 10.09%) in posterior maps.

### Distribution of fit errors

**Fig 3:**
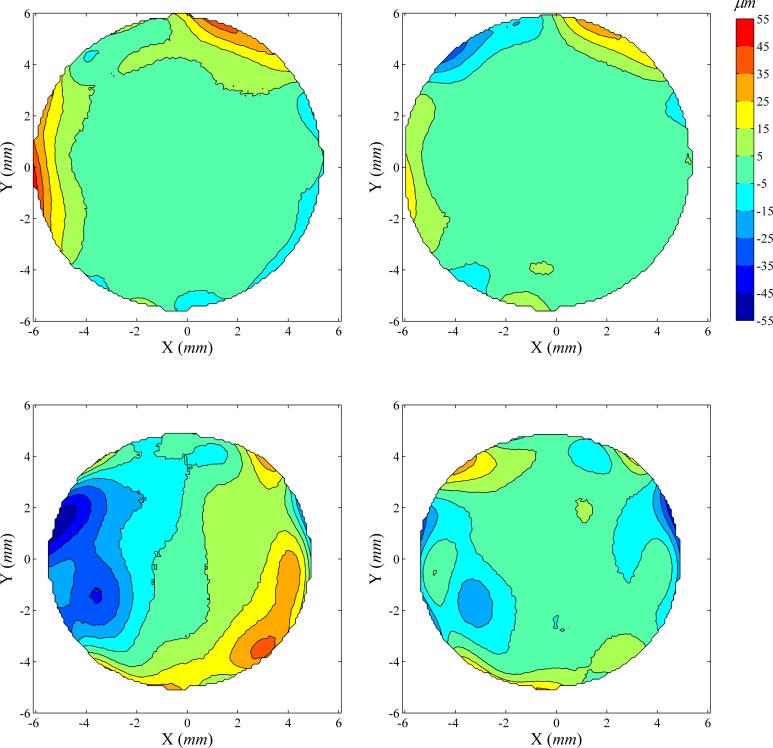
Distribution of elevation difference between successive corneal topographies before and after elimination of misalignment using ICP algorithm: Case 1. The first row shows contour maps of elevation difference in the common region of two successive anterior corneal topographies before (left) and after (right) elimination of misalignment using ICP algorithm, while the second row shows corresponding contour maps for posterior topographies. The four contour maps share the same colour scale (upright in μm). In this particular case, before ICP correction of misalignment, the RMS of fit error is 13.53 μm for both anterior and posterior surfaces, considered simultaneously, reduced to 7.24 μm (46.49% reduction) following the ICP correction.

**Fig 4:**
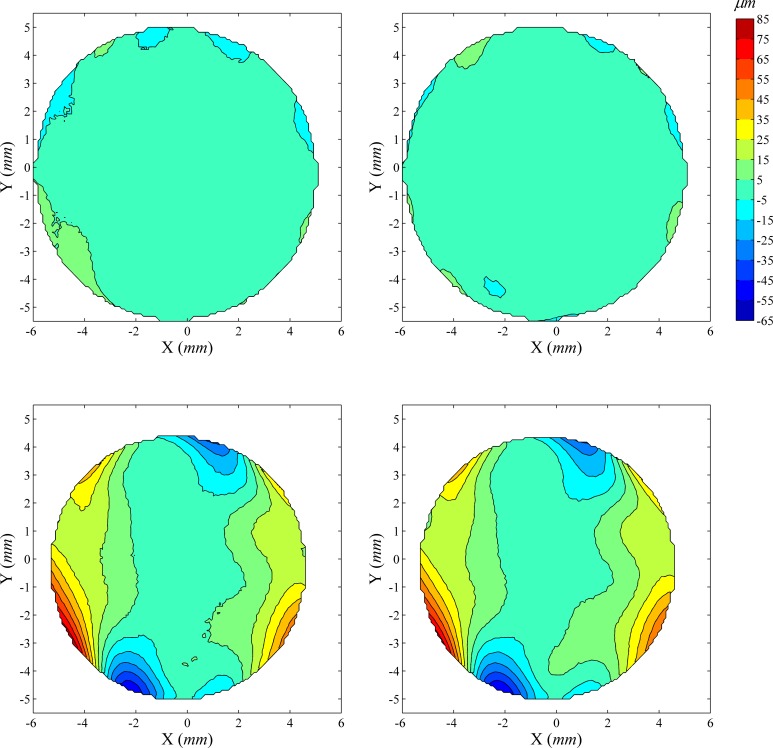
Distribution of elevation difference between successive corneal topographies before and after correction for misalignment: Case 2. Before ICP correction, the RMS of fit error was 12.07 μm for both anterior and posterior surfaces, considered simultaneously, and reduced by only 0.78% to 11.98 μm following correction.

The distribution of fit errors across map area was examined before and after ICP correction of misalignment. Two typical cases are shown in Figs 3 and 4. Fig 3 shows a case where ICP correction has improved the repeatability fit error significantly (reduction of 46.5%, from 13.53 μm to 7.24 μm), while Fig 4 shows a case with little error reduction (0.78% from 12.07 μm to 11.98 μm). In both cases, as well as all other tested cases, the fit errors for both anterior and posterior surfaces increased from the central to the peripheral region. After ICP correction, the central area with small fit error increased in size, especially in cases with large overall reductions in fit error (Fig 3).

### Association between combined misalignment parameter and map fit quality

**Fig 5:**
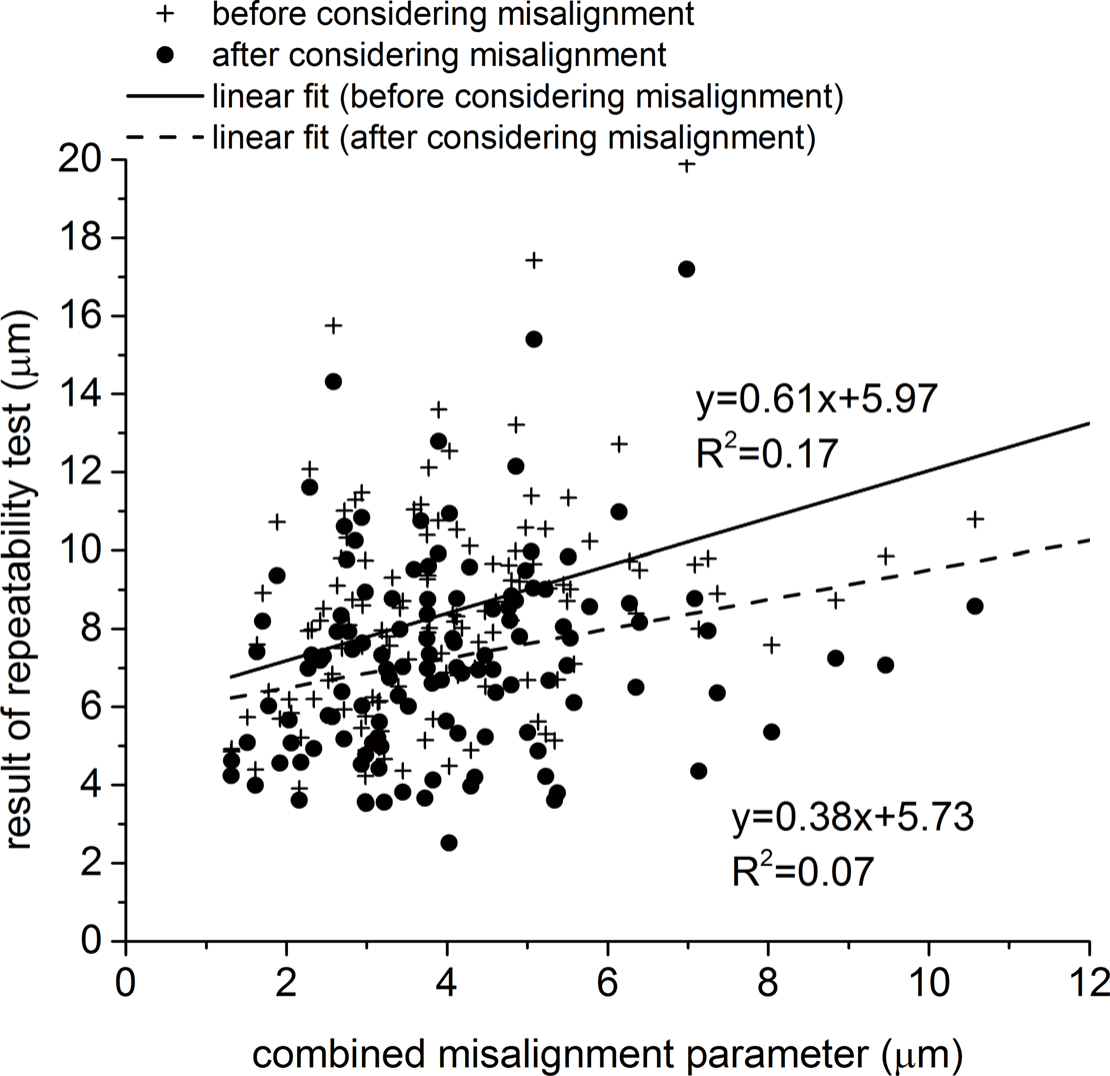
Correlation between the combined misalignment parameter and the RMS of error of fit within pairs of topography maps before and after correcting for misalignment. Anterior and posterior maps were considered simultaneously in both estimating and correcting for misalignment.

**Fig 6:**
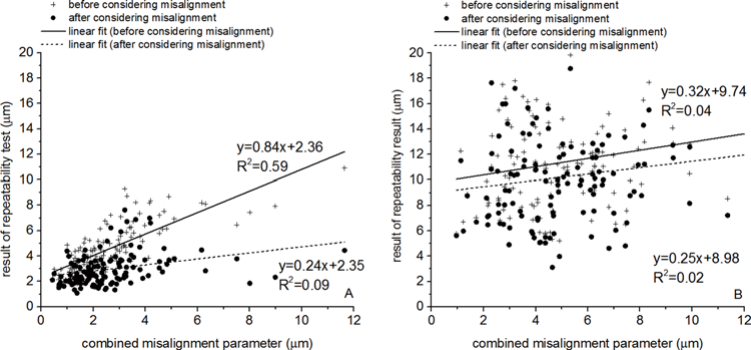
Correlation between the combined misalignment parameter and the RMS of error of fit within pairs of topography maps before and after correcting for misalignment. Anterior and posterior maps were considered simultaneously in estimating misalignment components but the effect of correcting for misalignment was tested in (a) anterior and (b) posterior maps separately.

Fig 5 shows the correlation between the CM, that is intended to combine the effect of all 6 misalignment components, and the RMS of fit error between pairs of maps, both before and after applying the ICP process to correct the second maps for misalignment. As expected, the correlation between the CM and the RMS of fit error reduced after correcting for misalignment, however, the effect was weak with limited reductions in correlation gradient from 0.61 to 0.38 and in the coefficient of determination (R^2^) from 0.17 (P = 0.00) to 0.07 (P = 0.00). This small effect and the low initial values of correlation gradient and R^2^ indicate that the ICP was less successful than expected in estimating and correcting for misalignment. This outcome could be a result of the significantly lower accuracy of posterior maps relative to anterior maps, and the fact that our method necessitated the simultaneous consideration of both sets of maps when using the ICP algorithm. In order to assess the effect of including posterior maps in the analysis, the results presented in Fig 5 were separated for anterior and posterior maps and presented separately in Fig 6A and 6B, respectively. These figures show that while both map sets have the same values of misalignment components, as estimated by the ICP, the anterior maps exhibited a stronger correlation between the CM and the RMS of fit error within map pairs. For anterior maps, the gradient of CM–RMS error was 0.84 and the R^2^ was 0.59 (P = 0.00), both reducing significantly after correcting for misalignment to 0.24 and 0.09 (P = 0.00), respectively. On the other hand, posterior maps, which still had the same misalignment components, showed initially a lower gradient of 0.32 and R^2^ of 0.04 (P = 0.02), both reducing slightly after correcting for misalignment to 0.25 and 0.02 (P = 0.06), respectively.

**Fig 7:**
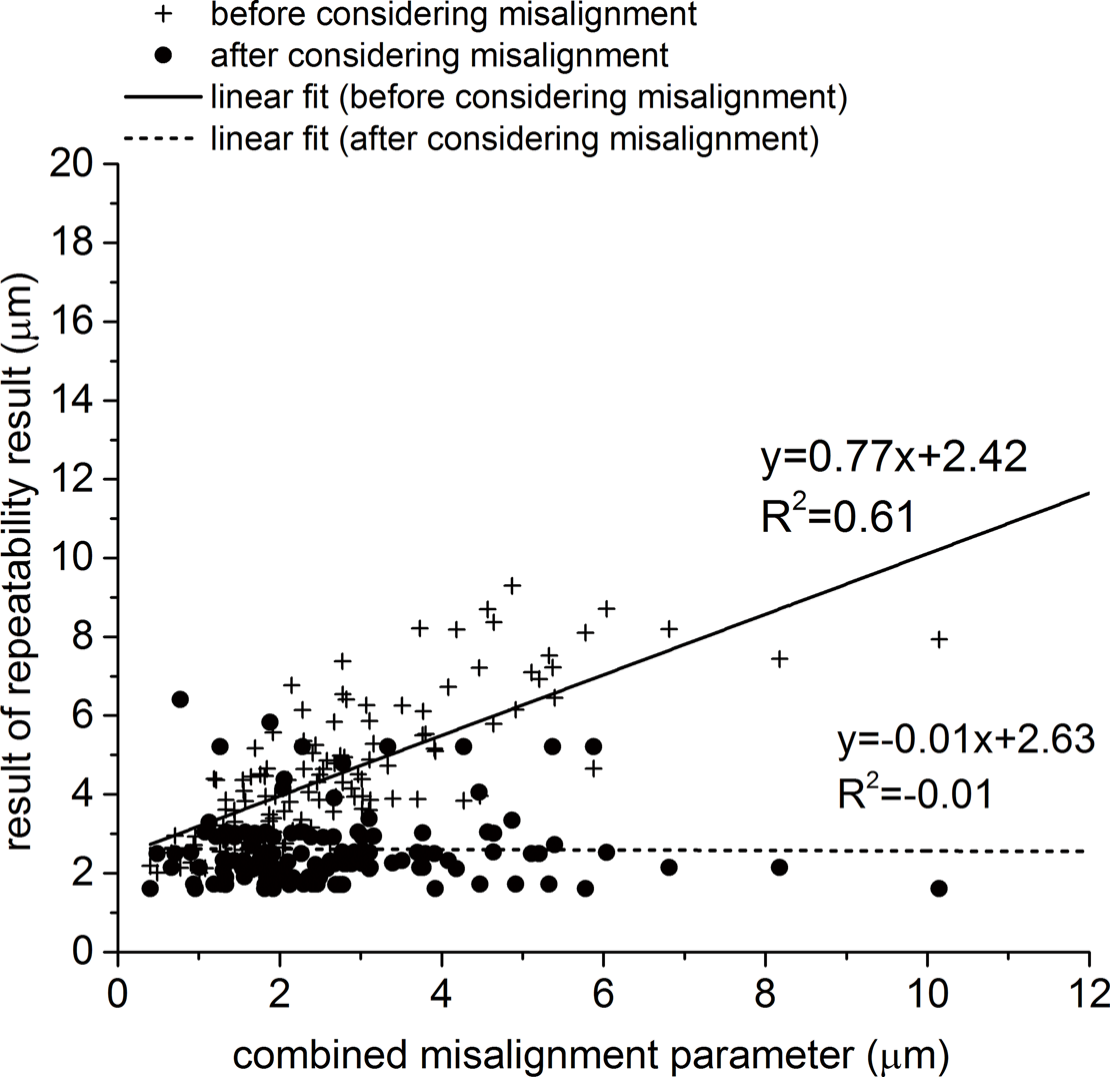
Correlation between the combined misalignment parameter and the RMS of error of fit within pairs of topography maps before and after correcting for misalignment. In this exercise only anterior maps were considered through whole process of estimating and correcting for misalignment.

Further, an additional test has been conducted, in which anterior maps were considered separate from posterior maps throughout the whole process of estimating and correcting for misalignment. Without considering posterior maps, anterior maps exhibited strong correlation between the misalignment parameter and the RMS of fit error, in the form of a gradient of 0.77 and R^2^ of 0.61 (P = 0.00). Both reduced considerably after correcting for misalignment to -0.01and -0.01 (P = 0.89), respectively (Fig 7).

## Discussion

Instruments used in clinics should give accurate and repeatable readings. Repeatability, defined as the consistency between readings on the same instrument under the same conditions, should be high, and same or very similar results should be obtained if the measuring instrument produces repeatable data. A common feature of most of the current videokeratography (VK) systems is that their maps are viewer-centered representations of topography, the accuracy and reliability of which are influenced by fixation lags and eye movements. Large misalignments are usually rejected by topography devices or compensated during data acquisition [18, 22] but smaller misalignments may be unavoidable. In spite of their small magnitude, these misalignments may affect the accuracy of biometric parameter estimates such as corneal curvature and asphericity in both anterior and posterior surfaces and makes comparisons between successive maps, either taken in the same setting or over time, less reliable. This study attempts to address this important point as it introduces a method, based on the ICP algorithm, to estimate, and eliminate the effect of, misalignment between successive topography maps and hence improve the repeatability of topographic data.

The ICP algorithm, originally proposed to align 3D surfaces, has become the dominant method for matching maps collected for the same object but from different viewpoints [23]. The use of ICP algorithm to estimate and correct for misalignment in corneal topography maps is believed to be more straightforward and potentially more computationally efficient than earlier methods such as those developed by Tobias et al [8] and Franklin et al [24, 25]. Unlike our method, Tobias’s method did not take into account that, concomitant with gaze changes, the area of measurement changed from one map to another, leading to potential inaccuracies. On the other hand, Franklin and co-workers presented a technique based on the least squares method to match central and peripheral maps in which attention was limited to fitting points on the peripheral maps to points on the central maps, rather than fitting the surfaces together. Their technique further allowed only for relative shifts but not rotations of maps, and considered only the parts of the peripheral maps outside the central map region, rather than using the whole peripheral maps in producing the final overall corneal map. Franklin’s technique is also based on the differences in axial radii of curvature as the alignment criterion, the measurement of which depends on the VK axis and represents curvatures only along the meridians while ignoring shape properties in other directions [26]. In the present study, (1) the ICP algorithm was used instead of the least squares method to improve computational efficiency, (2) focus has been on fitting surfaces rather than sets of points, (3) both translation and rotation of the peripheral surface was enabled, and (4) elevation rather than curvature data were used. A potential drawback of the method is its reliance on an initial, estimated position of the moving surface. However, the fact that misalignment between topography maps is likely to be small makes it possible to start the calculations while assuming all misalignment components have zero values.

The ICP algorithm is expected to be more suitable for matching elevation maps than curvature maps. Since curvature measurements are videokeratograph axis dependent and represent curvatures only along the meridians [22], similar curvature maps may depict different shapes if their reference axes were different, making the ICP algorithm potentially unsuitable in this case. An exception to this rule is when the curvature map becomes independent of the reference axes as in the less-commonly used corneal topography system Cassini (i-Optics, Netherlands), in which curvature data is obtained from a grid style reflection method. Additionally, elevation maps may present another problem if the shapes under consideration were too smooth and featureless, leading to ambiguous alignment. However, the non-spherical form of the cornea and its lack of rotational symmetry enable exclusion of this possibility and provide confidence in the misalignment estimates.

The effectiveness of the ICP algorithm in estimating misalignment between successive maps was assessed in the study before using it on the clinical data. The assessment relied on a typical topography map, randomly selected from the clinical database. The map was duplicated with the copy subjected to sets of known misalignment components. The maps (original and shifted copy) were then re-aligned using the ICP algorithm and the misalignment estimations were compared with the pre-set values. The results of this exercise provided strong evidence of the effectiveness of the algorithm with the max error of fit between the maps being slightly above 1% of the pre-set values.

Correction of misalignment in the clinical database led to mean reductions in elevation differences from 8.46±2.75 μm to 7.28±2.58 μm, when considering anterior and posterior maps simultaneously. However, since anterior maps were expected to have higher accuracy compared to posterior maps (and hence lower noise levels), the two sets of maps were considered separately, leading to mean reductions in elevation differences from 4.58±1.84 μm to 2.97±1.29 μm (mean reduction of 35.13%) in anterior maps, and from 11.40±4.05 μm to 10.25±3.89 μm (mean reduction of 10.09%) in posterior maps. These results show that the relatively higher accuracy and the lower noise of anterior maps made the ICP algorithm more effective and the resulting improvement in repeatability more pronounced.

Column diagram plots showed that the misalignments (transformations determined by the ICP algorithm) between successive measurements had a normal distribution with an even dispersion around zero values. Further, most of the misalignment components were not significantly different from zero except for z_0_, for which, the 95% limit of agreement (95% LoA) was -3.22 μm to 2.33 μm, and was not high enough to have clinical significance. Additionally, the overlap area ratio between the first and second measurements was 98.17±0.22% and 98.00±0.20% for anterior and posterior surfaces after ICP process, respectively, which were quite high and represented the good repeatability of the Pentacam.

The errors of fit calculated at individual measurement points (i.e. the elevation difference between successive maps determined at each grid point) consistently grew from the corneal central region to the periphery. After the ICP correction of misalignment, the central area with smaller fit errors increased in size while the bigger values of fit errors in the periphery remained. It also can be seen that posterior maps, while showing the same pattern of error growth from the central region to the periphery as anterior maps, always exhibited larger values of fit errors.

The more pronounced fit errors in peripheral areas compared to central areas and in posterior maps compared to anterior maps may have been caused by optical distortion due to aberrations in the instrument’s measuring lens [17, 23], and is expected to have affected the values of misalignment between measurements, as estimated by the ICP algorithm. It can therefore be argued that the accuracy of topographic data and the repeatability of VK maps could be improved by utilising only the central regions of anterior maps when estimating misalignment.

A new parameter has been developed to combine the effects of individual misalignment components and enable quantifiable assessment of the relationship between misalignment and data repeatability. The parameter considered the effects of individual components on an idealised map with the average corneal dimensions in order to enable comparison between the effects of individual misalignment components and between the combined effects of whole components’ sets. Using the values of this parameter, there was a clear correlation between misalignment and the results of the repeatability tests, although this correlation is thought to have been affected by the inevitable data noise. The results also showed the better accuracy of data in anterior maps relative to posterior maps as has been illustrated earlier in the study.

The CM enabled a comparative assessment of the effects of individual misalignment component. For cases involving the following individual misalignments; (1) x_0_ = 1 μm, (2) y_0_ = 1 μm, (3) z_0_ = 1 μm, (4) α = 1 min (1/60 degree), (5) β = 1 min, and (6) γ = 1 min, CM had values of 0.314 μm, 0.322 μm, 1.000 μm, 0.734 μm, 0.731 μm and 0.004 μm, respectively. These results showed that translation along x and y axes led to similar overall misalignment effects and similar effects on repeatability, and the same was true for rotations around x and y axes.

The study attempted to evaluate the effects of possible misalignment between successive corneal maps on the accuracy of topographic data. Although the misalignment values were small, and in most cases not statistically different from zero, they led to considerable reductions in data repeatability and could therefore have an effect on the topography data guiding clinical decisions. The study showed that while topography maps suffered from data noise, the central cornea and the anterior surface had better repeatability than the peripheral cornea and the posterior surface. The study showed that the ICP algorithm was successful in estimating and eliminating the effect of misalignment, leading to improved repeatability and hence more confidence in topographic data, especially in the anterior surface. The simple application of the ICP and the resulting improvement in data repeatability suggest that misalignment between successive maps should be eliminated in applications where topographic data is important for clinical decision making.
